# Fabrication of Aluminum Oxide Thin-Film Devices Based on Atomic Layer Deposition and Pulsed Discrete Feed Method

**DOI:** 10.3390/mi14020279

**Published:** 2023-01-21

**Authors:** Shih-Chin Lin, Ching-Chiun Wang, Chuen-Lin Tien, Fu-Ching Tung, Hsuan-Fu Wang, Shih-Hsiang Lai

**Affiliations:** 1 Mechanical and Systems Research Lab, Industrial Technology Research Institute, Hsinchu 310401, Taiwan; 2 Department of Electrical Engineering, Feng Chia University, Taichung 40724, Taiwan

**Keywords:** thin film, buffer layer, aluminum oxide, atomic layer deposition, plasma-enhanced chemical vapor deposition, pulsed discrete feed method, mechanical stress

## Abstract

This study demonstrates the low-temperature (<100 °C) process for growing a thin silica buffer layer and aluminum oxide by atomic layer deposition (ALD) in the same reaction chamber. Heterogeneous multilayer thin films are prepared by a dual-mode equipment based on atomic layer deposition and plasma-enhanced chemical vapor deposition (PECVD) techniques. The pulse discrete feeding method (DFM) was used to divide the precursor purging steps into smaller intervals and generate discrete feeds, which improved the saturated distribution of gas precursors, film density and deposition selectivity. The experimental results show that the process method produces a uniform microstructure and that the best film uniformity is ±2.3% and growth rate is 0.69 Å/cycle. The thickness of aluminum oxide film has a linear relationship with the cyclic growth number from 360 to 1800 cycles. Meanwhile, the structural and mechanical stress properties of aluminum oxide thin films were also verified to meet the requirements of advanced thin-film devices.

## 1. Introduction

Aluminum oxide (Al_2_O_3_) is a promising optoelectronic material due to its optical, chemical and electrical properties. Aluminum oxide thin film has been used as the gate dielectric for transparent thin film transistors [1,2], the packaging of photovoltaic devices [3], the diffusion barrier layer of gas [4] and the surface coating of electrodes and photoelectrodes [5]. Al_2_O_3_ thin films can be prepared by different coating techniques, such as high power impulse magnetron sputtering (HiPIMS) [6], pulsed laser deposition (PLD) [7], electron beam evaporation [8,9,10], sol-gel [11] and atomic layer deposition (ALD) [12,13,14,15]. Among these techniques, atomic layer deposition (ALD) is a key process in the optoelectronic semiconductor industry. ALD has become one of the most promising methods for deposition and production of high-quality films. It has proved and shown its potential to meet various application requirements [16]. With the increase of the manufacturing scale of various optoelectronic semiconductor devices, the demand for ultra-thin film uniformity and the coating ability of complex 3D structures has also increased. ALD is a chemical self-limited deposition technique based on the continuous use of gaseous chemical processes. In most cases, the ALD reaction uses two chemical precursors that react with the surface one at a time in a sequential manner. In the chamber, the substrate is placed at a given temperature and pressure so that the material can be deposited layer by layer on the surface of the substrate [17]. As the manufacturing scale of various optoelectronic semiconductor devices increases, the need for ultra-thin film layer uniformity and the coating ability for complex 3D structures also increases. The ALD technique uses a unique self-limiting surface reaction to grow films with high step coverage and uniformity in a large area, in order to achieve excellent performance of ultra-thin nano film or atomic scale films. Because ALD technique has the characteristics of high density, high thickness uniformity, high step coverage, low temperature process and atomic level thickness control, it can not only provide filling capacity for ultra-thin material coatings, but also provide filling capacity for ultra-thin material coatings for micro-groove and pore structures, such as 3D geometric coating, with uniform thickness and a high aspect ratio structure.

The main reaction mechanism of atomic layer deposition is to grow the film layer-by-layer using the pulse generated from precursor gas or vapor, and then deposit to a substrate surface. The common method is to use two precursors and deposit them alternately on the reaction substrate. In the first step, functional group A is adsorbed on the first layer. Secondly, the functional group B can react with it and form monolayer AB. The reaction occurs between the functional groups of the precursor molecule at the surface. Thus, the monolayer can grow as a large-scale thin film layer through layer-by-layer reactions.

**Figure 1:**
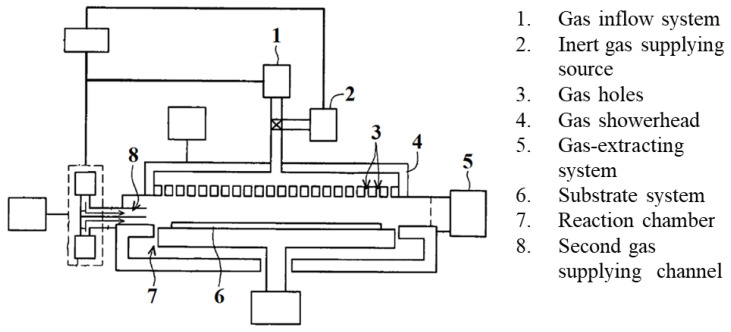
Schematic diagram of the cross-section of the dual-mode atomic layer deposition coating equipment.

A US patent has been obtained for a multi-mode thin film deposition apparatus and method of depositing a thin film [18]. The dual-mode atomic layer deposition coating equipment is as shown in Figure 1. This patent integrates the ALD technique and plasma-enhanced chemical vapor deposition (PECVD) in a single common chamber to achieve the purpose of producing heterogeneous thin film layer. It not only shortens the time for specimen transfer between chambers, but also reduces the potential pollution in the transfer process. In addition, the dual-mode equipment can meet the low temperature (<100 °C) process requirement, and cover multi-component films in the ALD and PECVD processes, making it suitable for applications in barrier layers and ladder structure coverage. Although the ALD technique can form dense thin films, it requires relatively more processing time due to the active chemisorption efficiency of the precursor molecules. Therefore, the discrete feeding method (DFM) can be controlled to enhance the coating thickness and atomic layer coating rate [19,20].

Although ALD has many advantages, there are still some inherent problems to be solved before its application in low-temperature manufacturing process, such as insufficient growth rate and unreliability of thin films. Unfortunately, the low productivity of ALD thin film is unavoidable because of its self-limiting behavior [21]. Besides, it is necessary to improve the mechanical stress of thin films grown at low temperature, which leads to the ability of insulating materials to withstand the applied electrical stress. This issue is very important for dielectric/insulating films in electronic devices. Therefore, this study demonstrates the ALD low-temperature process for a thin silica buffer layer and aluminum oxide used for thin film semiconductor devices. We also validated the research process for advanced thin-film devices by validating the surface profile, structural and mechanical stress properties of the aluminum oxide thin films.

## 2. Materials and Methods

**Figure 2:**
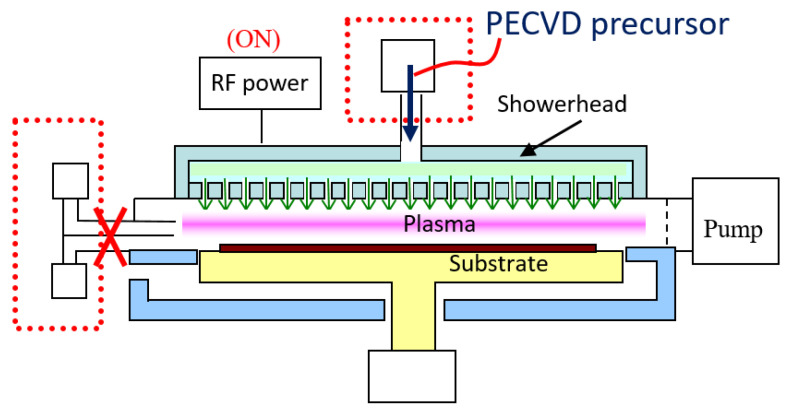
Schematic diagram of dual-mode plasma-assisted vapor deposition coating process.

**Figure 3:**
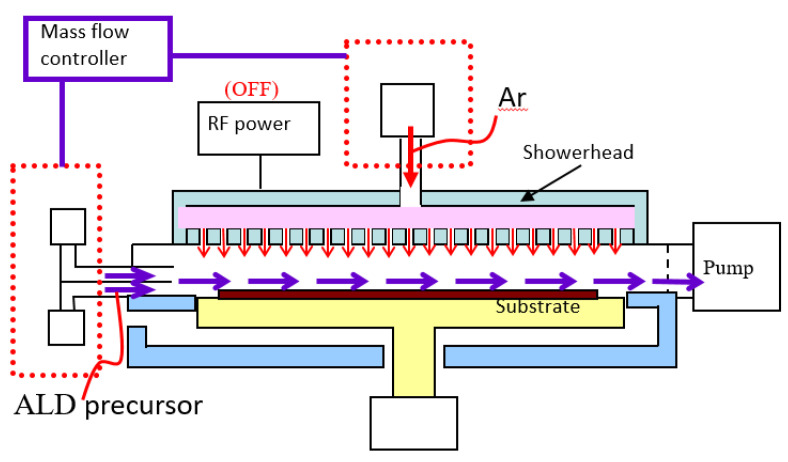
Schematic diagram of dual-mode atomic layer deposition coating process.

To fabricate aluminum oxide thin-film devices, the dual-mode deposition equipment is used for the thin film process in this study. The schematic diagram of the dual-mode atomic layer deposition coating equipment is shown in Figure 1. The system’s structure is mainly composed of a process vacuum chamber, plasma-enhanced dissociation vapor deposition showerhead, radio frequency power system, vacuum pumping automatic pressure controller (APC), substrate carrier module and process gas source components. The space between the gas showerhead and the substrate carrier module is the thin film deposition reaction chamber, and the substrate is placed above the substrate carrier in a dual-mode deposition cavity. The vacuum pumping automatic pressure control system vacuumizes the chamber and adjusts the gas pressure required by the process. The vacuum pump takes away the process by-products and unreacted materials. Furthermore, the plasma-enhanced chemical vapor deposition (PECVD) method is different from the atomic layer deposition technique in terms of hardware applicability. PECVD is a chemical vapor deposition process used to deposit thin films from a gas state (vapor) to a solid state on a substrate. PECVD utilizes an electrode to volatilize precursors. This technique has been widely used to obtain high quality thin films at low substrate temperatures. The PECVD module needs to uniformly distribute the mixed process gas through the gas showerhead, and generates plasma to decompose the processing gas in order to form the precursor reactants for coating. It achieves uniform film deposition through chemical reaction. However, the ALD technique is expected to allow the gas precursors to saturate and adsorb on the process substrate within the lower cycle time. Figure 2 shows the dual-mode process for the atomic layer deposition and plasma-enhanced chemical vapor deposition module. When the air inlet system for atomic layer deposition is shut-off, the gas required for the PECVD process is passed from the showerhead into the process chamber, and the power system generates the plasma for thin film deposition. When the equipment is switched to the ALD to grow metal oxide films, the precursor is fed in a horizontal side channel flow to guide the direction of the flow gas into the processing cavity, so as to carry out the atomic layer coating of the airflow field as shown in Figure 3. There are several hybrid CVD methods that combine two or more film coating techniques and take advantage of each technology.

**Figure 4:**
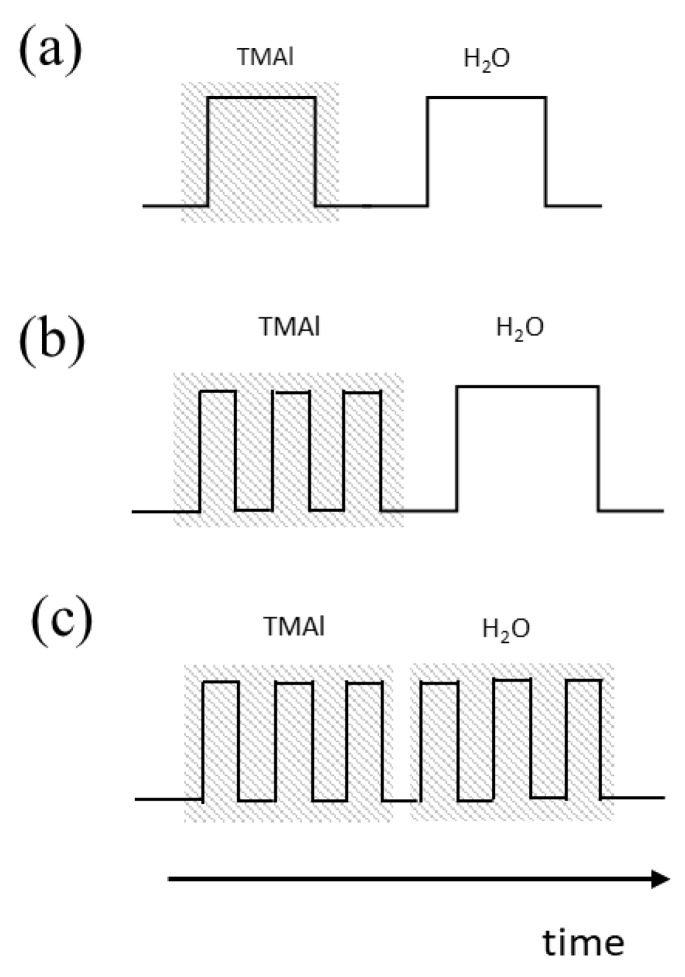
The dual-mode atomic layer deposition by pulsed discrete feed method via time differentiation. (**a**) Original discrete feed method; (**b**) the feeding interval of the organic metal precursor remains unchanged; (**c**) feed interval shortened at the same ratio.

In the manufacturing process, plasma-assisted chemical vapor deposition (PECVD) was carried out first, and the 2-inch B270 glass substrate was coated with an ultra-thin buffer layer of silicon dioxide (SiO_2_), using silane and O_2_ as reactants at 80 °C and 300 W. After finishing the buffer layer, the ALD technique was used to grow aluminum oxide films. The process used pulsed discrete feed to control the time of precursor feed, and was divided into smaller time intervals, so that the precursors enter the cavity in batches to generate discrete feed, which improves the coating properties and enhances the compactness and deposition selectivity of the film. For the Al_2_O_3_ coating process, trimethylaluminum (TMA) was used as the precursor and water molecules as the oxygen source. Different control methods can be compared. The original discrete feed method is shown in Figure 4a. The difference is only that the material feeding interval of the organic metal precursor is unchanged, as shown in Figure 4b, or the feeding interval of the organic metal precursor and the water molecule precursor at the same ratio is reduced, as shown in Figure 4c. TEM measurement (FEI, Talos F200X) identifies the microstructure and phase of the thin film.

The mechanical residual stresses of thin films are affected by the different processes and coating parameters, which result in different packing densities. Common thin film mechanical property tests include adhesion, nano-indentation, hardness, residual stress and biaxial modulus, etc. This work evaluates the residual stress in thin films after growing Al_2_O_3_ film on the B270 glass substrate by atomic layer deposition technique. The residual stress in Al_2_O_3_ thin films is measured by a Twyman-Green interferometer combined with fast Fourier transform (FFT) method [22,23,24], which are excellent in both measurement accuracy and operation speed. Most of the thin films exhibit residual stress with different levels, and different process conditions, coating materials, and even the type of substrates. are related to the residual stress in thin films. According to the mechanical stress properties of thin films, the residual stress can be divided into two types, tensile stress and compressive stress. To maintain mechanical equilibrium, the net force and bending moment on the film/substrate cross-section are required to be zero. If the growing film initially shrinks relative to the substrate, the film is constrained and stretches, while the substrate accordingly contracts. Thus thin films containing internal tensile stresses bend the substrate concave upward. This is a tensile stress, which is defined as a positive value. Similarly, internal compressive film stresses bend the substrate convex outward. This is a compressive stress, numerically defined as negative.

In this study, fast Fourier transform (FFT) method is used to analyze the interferograms, and then the phase of the coated substrate and the film surface is restored to obtain the surface profile of thin film. Then the radius of curvature before and after coating is obtained by the curve fitting, and the residual stress value of thin film can be determined by using the modified Stoney formula [25,26].

(1) $$\sigma =\frac{E_{s}\cdot t_{s}^{2}}{6\left(1-v_{s}\right)t_{f}}\left(\frac{1}{R_{2}}-\frac{1}{R_{1}}\right)$$

where *σ* is the residual stress of thin film, *E_s_* is the Young’s modulus of the substrate, *v_s_*. is the Poisson’s ratio, *t_s_* is the thickness of the substrate, *t_f_* is the thickness of the thin film, and *R*_1_ and *R*_2_ are the radii of curvature before and after film deposition. Thus, the residual stress in thin film can be obtained by the modified Stoney formula.

## 3. Results and Discussion

In general, the ALD growth mode of the metal oxide films is based on the reaction through the exchange of ligands between the precursor and oxygen source molecules to form a surface. The growth rate of ALD films increases with the feed time of the metal precursor in the sub-saturated region, and when the precursor dose is high enough to completely cover the active sites on the film or substrate surface, saturation occurs at a specific feed time, which is considered as the growth-saturated feed time and condition. In the early stage, several important factors could affect the growth rate for the ALD film saturation. The following steps are involved: first, the steric hindrance effect due to decomposition of some neighboring molecules during the precursor feeding period [27]; second, the active site in the lower layer shielded by the physisorbed precursor molecules. Third, reaction by-product molecules shielding the adsorption sites and blocking the active chemisorption of the precursor molecules. By using the pulsed discrete feed method (DFM), the aforementioned inherent limitations of atomic layer film growth can be overcome [28]. The main method is by dividing one complete step into several shorter feed/cut-in purge steps during the precursor feed, which can open the voids of active sites on the surface by continuous sweeping and enable efficient filling of molecules during the feed step to achieve complete surface saturation and coverage without wasting precursors.

### 3.1. Pulsed Discrete Feed Method

**Figure 5:**
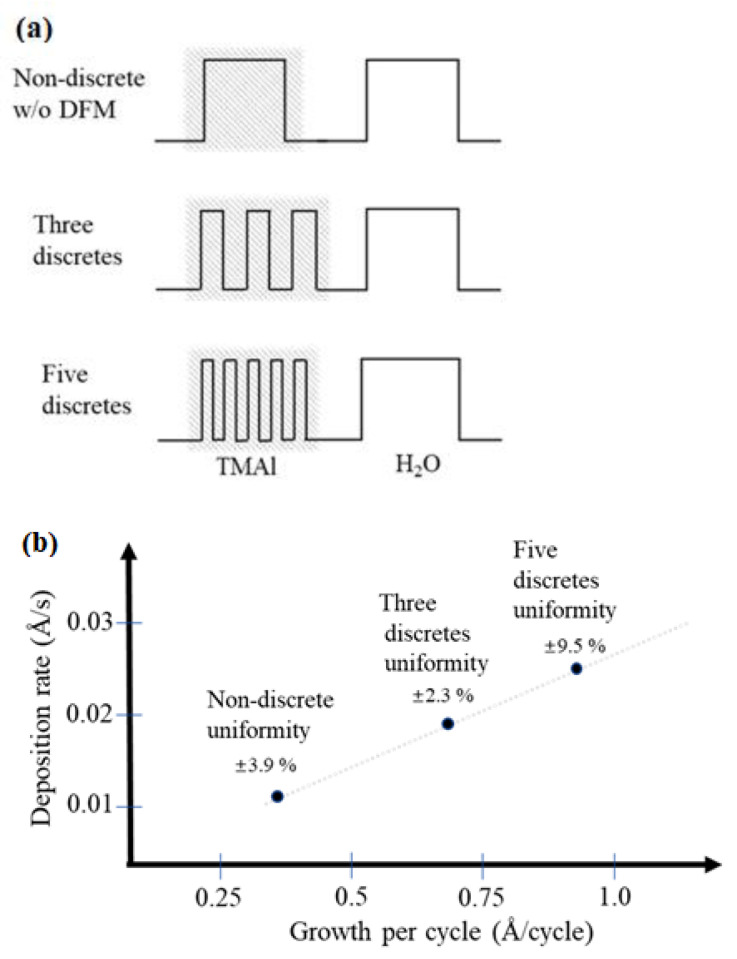
Pulsed discrete feed method for dividing metal precursors only. (**a**) Pulsed discrete feed time dividing diagram and (**b**) deposition rate versus grown rate.

In this study, a pulsed discrete feed method (DFM) was used to divide the precursor purge step into smaller time intervals and enter the cavity in batches, resulting in discrete feed. Thus, the saturation distribution of gas precursors, thin film compactness and deposition selectivity can be improved. When the thin films were not deposited by the pulsed discrete feed method, this is the non-discrete mode, in which the uniformity of the aluminum oxide films was ±3.9%, the deposition rate was 0.66 Å/min and the growth rate was 0.34 Å/cycle. Using the pulse discrete feeding method, the non-discrete feed of the corresponding TMA metal precursors of the above film was replaced by a three-discrete or five-discrete feed with a smaller interval. Film formation rate and thickness uniformity were compared. The illustrative diagram of the feed time in pulse division is shown in Figure 5a, while Figure 5b shows the relationship between the film growth thickness per cycle and the film deposition thickness per second. When TMA feed is divided into three equal smaller portions, the best film uniformity is ±2.3%, the deposition rate is 1.38 Å/mi, and growth rate is 0.69 Å/cycle. This shows that, compared with the non-pulse discrete feed method, the film uniformity is significantly improved and good film uniformity is obtained. In addition, the TMA feed is divided into five equal parts, so the film uniformity obtained is ± 9.5%, the deposition rate is 1.44 Å/min and growth rate is 0.92 Å/cycle. Therefore, the more TMA feed is divided, the higher the coating rate and the higher the non-uniformity. It is obvious that the optimized parameters can be obtained by dividing the TMA precursor feed into three equal parts. If the number of discrete feeds is too large, although the steric hindrance effect is improved, the purge speed is too fast, so the adsorption growth filling is insufficient and an improved self-limiting growth can not be obtained.

### 3.2. Experimental Conditions and Structural Characteristics

**Figure 6:**
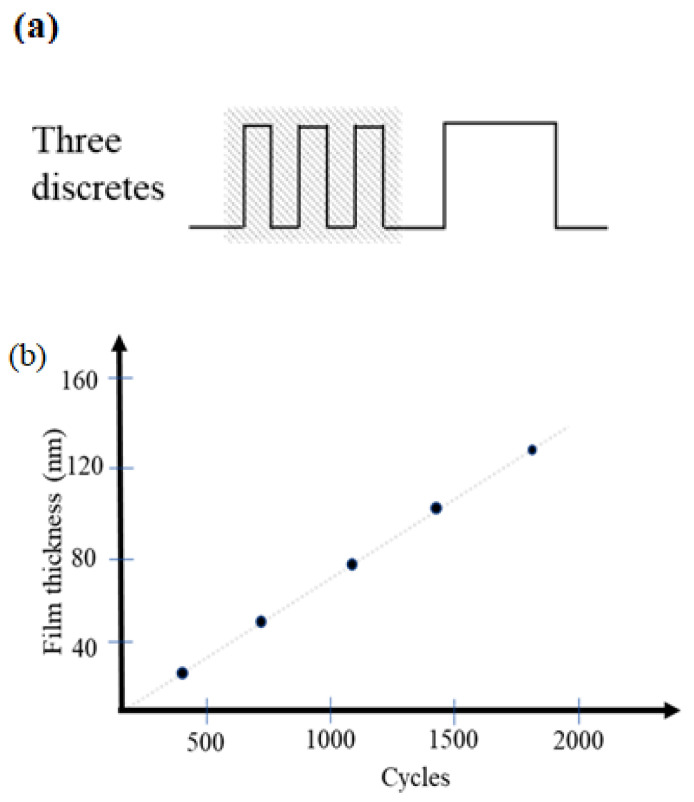
(**a**) The TMA feed is divided into three equal parts; (**b**) Relationship between the number of coating cycles and the thickness of aluminum oxide films.

**Figure 7:**
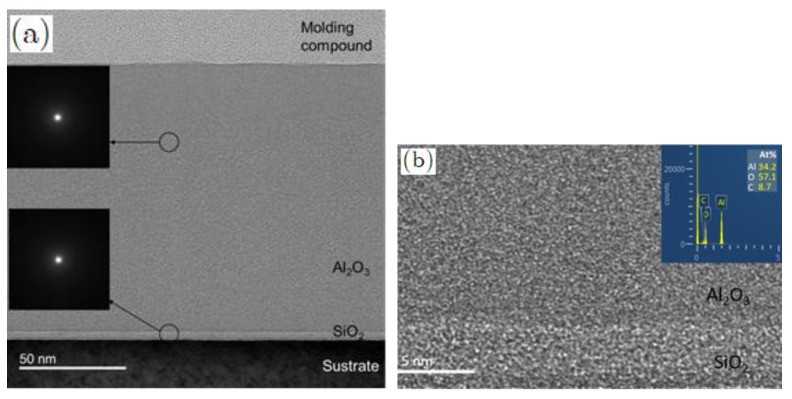
(**a**) TEM cross section image; (**b**) EDS spectrum of the Al_2_O_3_ thin film with 1800 cyclic growth.

TMA precursor material feed is divided into three equal portions, as shown in Figure 6a. The aluminum oxide thin film was coated on B270 glass substrates at process temperature of 80 °C. The TMA pulse time is 2 s and the cut-in purge time is 3 s; at the same time, the H_2_O pulse time is 6 s and its cut-in purge time is 10 s. Then the number of the coating cyclic growth was multiplied by 360, 720, 1080, 1440, 1800 cycles, and the samples were denoted as A1, A2, A3, A4 and A5, respectively. Table 1 shows the experimental conditions and film thickness for TMA as precursors for aluminum oxide thin films. The overall coating film cyclic growth number and aluminum oxide film thickness are in a linear relationship, as shown in Figure 6b. The transmission electron microscope (TEM) was used to identify the phase and microstructure of thin films. The specimens with thickness of about 200 μm were carefully polished down to 100 μm with the help of diamond paste. After polishing on both faces, it was noted that there existed a difference in thickness of approximately 20 μm. The residual thickness thus obtained in the center was approximately 10 μm. The specimen was then ready to undergo ion thinning. In addition, energy dispersive X-ray spectroscopy (EDX) was performed during the scanning electron microscope (SEM) examination of such samples. Figure 7a shows the cross-section image of the microstructure of the thin film grown at 1800 cycles. The EDX spectrum demonstrated the nano/atomic level structure. The O:Al ratio of the Al_2_O_3_ film was determined to be approximately 3.3: 2 to confirm the formation of approximately stoichiometric Al_2_O_3_ thin film, as shown in Figure 7b. The 10 nm SiO_2_ buffer layer was coated on the substrate by PECVD. Then, the dense Al_2_O_3_ thin films were continuously grown on the buffer layer. Since no diffraction peaks were observed by X-ray diffraction (XRD), the multilayer thin film showed an amorphous structure.

### 3.3. Surface Profile and Residual Stress Measurement

**Figure 8:**
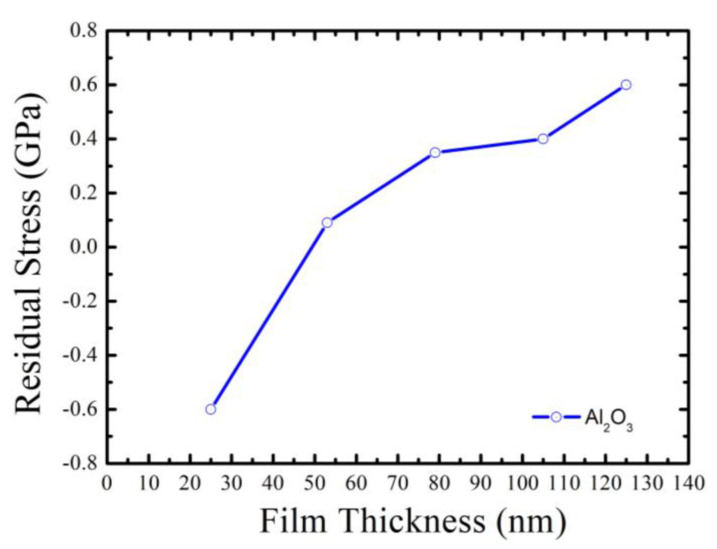
The relationship between residual stress and deposition thickness of Al_2_O_3_ thin film.

**Figure 9:**
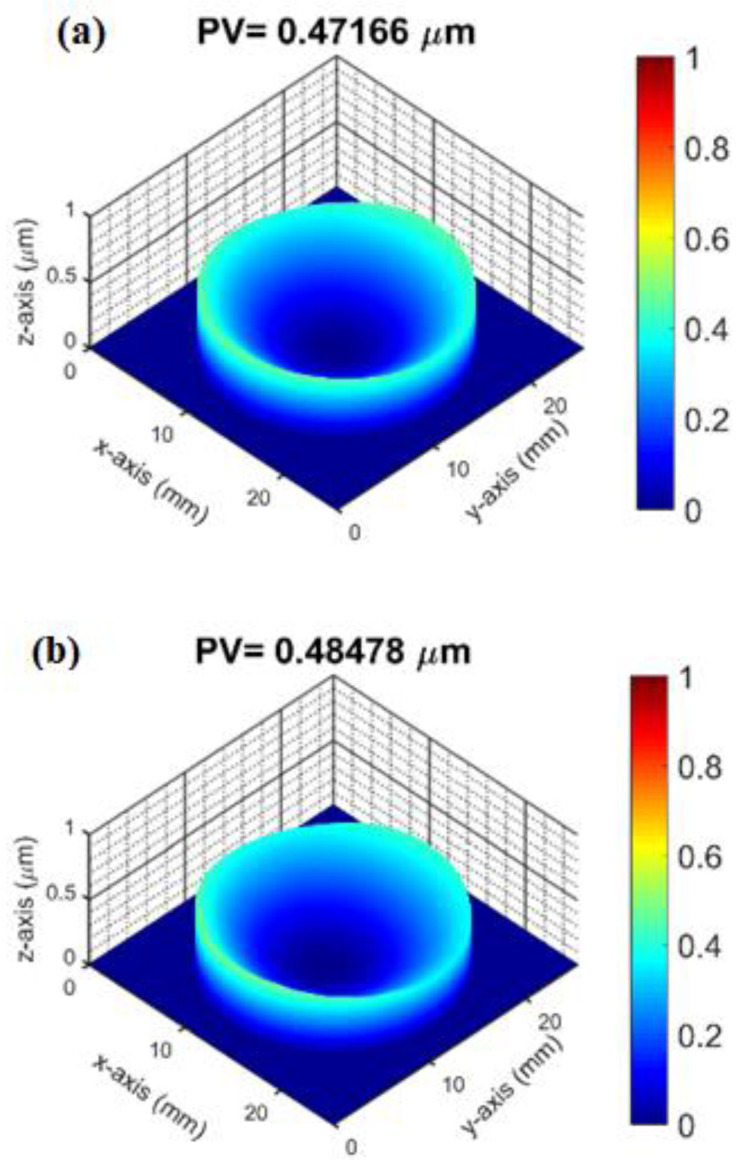
(**a**) Three dimensional profile of substrate before coating and (**b**) three dimensional profile of Al_2_O_3_ deposition thickness of 26 nm.

**Figure 10:**
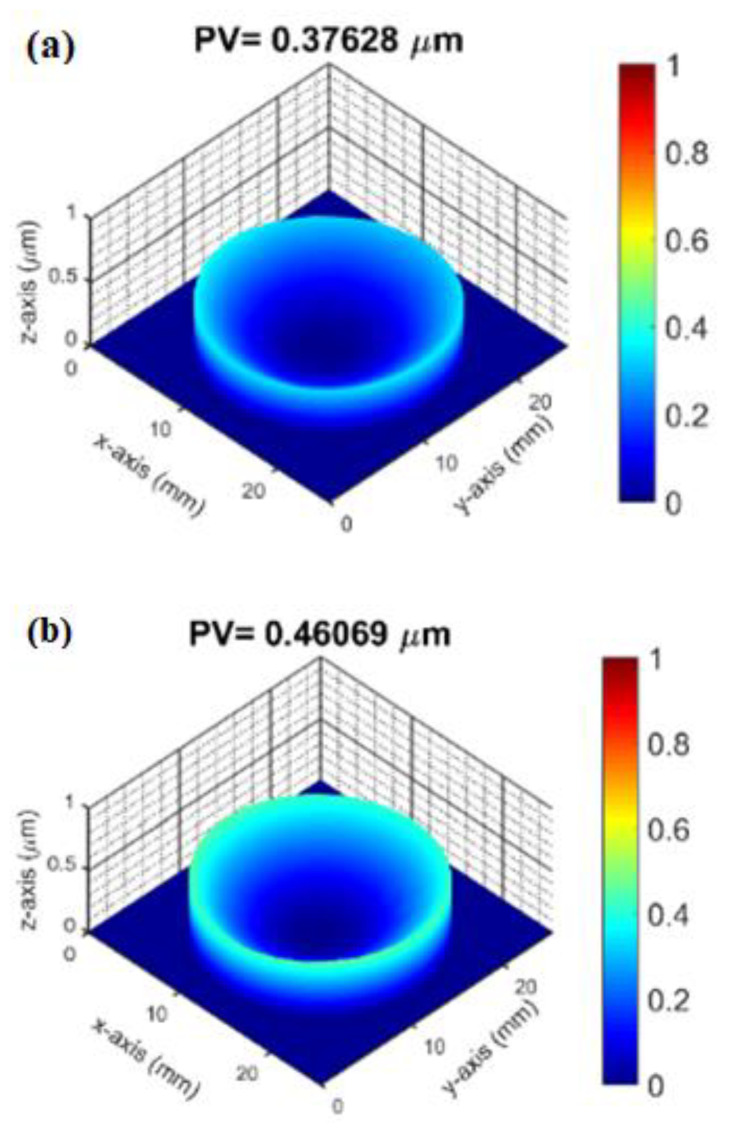
(**a**) Three dimensional profile of substrate before coating and (**b**) three dimensional profile of Al_2_O_3_ deposition thickness of 126 nm.

As can be seen from Figure 8, when the thickness of Al_2_O_3_ thin film is 26 nm, it shows a state of compressive stress. Compared to the three-dimensional profile of the substrate before coating and the three-dimensional profile of the Al_2_O_3_ film deposited with thickness of 26 nm, the peak-to-valley (PV) slightly increases from 0.472 μm (Figure 9a) to 0.485 μm (Figure 9b). As the thickness of the process increases, the Al_2_O_3_ film changes from a compressive stress to a tensile stress at a thickness of 52 nm. As the film thickness increases from 52 nm to 126 nm, it is found that the tensile stress of the Al_2_O_3_ film has an increasing trend. If we examine the three-dimensional profile of Al_2_O_3_ deposition with a thickness of 126 nm, the peak valley value (PV) increases from 0.376 μm (bared substrate) to 0.461 μm, which indicates that the surface after thin film coating becomes more concave (i.e., the state of tensile stress), as shown in Figure 10a,b. In addition, some studies have shown that the residual stress state of the thin films is related to their microstructure. The result reveals that as the thickness of the Al_2_O_3_ thin film increases, the tensile effect will reduce the packing density of the thin film and show a tensile stress. This tensile effect gradually increases with the deposition thickness, and makes the tensile stress the dominant stress in the film, leading to the transformation of the compressive stress in Al_2_O_3_ thin film into the tensile stress. Krautheim et al. [29] reported that the residual stress after deposition of Al_2_O_3_ film is generally in a tensile state, and the volume reduction caused by phase transformation is the main reason for the increase of tensile stress. The results of residual stress measurement are consistent with those reported in this literature.

## 4. Conclusions

The low-temperature (<100 °C) atomic layer deposition coating equipment is a breakthrough compared with the original planar coating and single continuous precursor feed method. We adopt the ALD technique and pulsed discrete feed method to prepare Al_2_O_3_ thin film. The results show that the coating rate increases with the number of discrete cycles from the test data of planar samples, which also indicates that the steric hindrance is improved. In particular, the pulsed discrete feed method divides the precursor sweep time into smaller intervals, which is suitable for introducing different precursors between different steps. This method can achieve a multi-component film coating effect in a very thin film layer, and improve the coating rate and film compactness. Regarding the residual stress of the Al_2_O_3_ thin film, the measurement results show that with the increasing of the ALD process thickness, the Al_2_O_3_ thin film changes from a compressive stress state to a tensile stress state. When the thickness increases from 52 nm to 126 nm, the result shows that the tensile stress of Al_2_O_3_ thin films has an increasing trend. This is helpful in controlling and improve the mechanical stress of Al_2_O_3_ thin films. The above results also reveal that Al_2_O_3_ thin films could be considered as a functional material for micro-optoelectronic devices.

## Figures and Tables

**Table 1:** Experimental conditions and film thickness for TMA as precursors for aluminum oxide thin films.

Sample No.	Cycles	Thin Film Thickness (nm)
A1	360	26
A2	720	52
A3	1080	79
A4	1440	107
A5	1800	133

## Data Availability

Not applicable.
